# ASO Author Reflections: Looking Beyond Imaging—Multimodal Response Assessment after Modern Neoadjuvant Therapy for Pancreatic Ductal Adenocarcinoma

**DOI:** 10.1245/s10434-026-20107-3

**Published:** 2026-06-29

**Authors:** Paul H. McClelland, Syed A. Ahmad

**Affiliations:** Division of Surgical Oncology, Department of Surgery, University of Cincinnati Medical Center, Cincinnati, OH USA

## Past

Pancreatic ductal adenocarcinoma (PDAC) remains among the most lethal solid malignancies, and surgical resection offers the only path to cure, yet long-term survival is consistently undermined by early recurrence and occult metastatic spread. Against this backdrop, neoadjuvant therapy (NAT) has become central to the management of resectable and borderline-resectable PDAC, with the aims of treating micrometastatic disease, testing tumor biology, refining patient selection, and downstaging locally advanced tumors.^1^ Interpreting response to NAT, however, remains a persistent challenge with direct consequences for surgical decision-making, as continued therapy in the absence of a clear treatment signal raises concern for loss of the window for potentially curative resection. The three domains most often used to gauge treatment effect (radiographic response by Response Evaluation Criteria in Solid Tumors (RECIST) scoring, biochemical response by serum (carbohydrate antigen 19-9) levels, and histopathologic response by tumor regression grading) each carry recognized limitations, and their prognostic relevance and concordance with oncologic outcomes remain incompletely defined.^2–4^

## Present

This study evaluated all three response domains in a contemporary single-institution cohort of 166 patients with resectable or borderline-resectable PDAC treated with multiagent NAT between 2008 and 2024.^5^ Radiographic response (RECIST 1.1 CR/PR) was uncommon, occurring in only 27% of patients, whereas biochemical response (≥ 45% CA 19-9 decline, 83%) and histopathologic response (College of American Pathologists score 0–2, 74%) were frequent. Notably, even among resected patients with radiographically stable or progressive disease (RECIST 1.1 SD/PD), most still demonstrated biochemical response (88%) and at least partial histopathologic response (68%). Neither radiographic nor biochemical response was significantly associated with progression-free or overall survival, whereas histopathologic response was independently associated with improved progression-free survival (aHR 0.57, 95% CI 0.34–0.96). Isolated unresectable local progression during NAT was rare (*n* = 2), although broader treatment-related attrition (predominantly attributable to systemic progression) remained clinically meaningful. Collectively, these findings indicate that the absence of radiographic response frequently masks underlying treatment effect and should not by itself be equated with treatment failure.

## Future

These results support a paradigm in which operative decision-making after NAT does not hinge on imaging response alone: absent objective disease progression or anatomic unresectability, the lack of radiographic regression should not unilaterally preclude resection. Realizing a new, holistic clinical model for PDAC will instead require composite response criteria that integrate radiographic, biochemical, histopathologic, performance-status, and emerging molecular measures, prospectively validated against recurrence and survival. On the imaging axis, quantitative radiomics and machine-learning models such as texture analysis of pretreatment/posttreatment cross-sectional studies may detect therapy-induced change that escapes conventional size-based criteria. On the biochemical axis, longitudinal blood biomarkers such as circulating tumor DNA (ctDNA) offer a dynamic, minimally invasive readout of residual disease that could complement or eventually supplant serial serum CA 19-9. Importantly, the meaning of response itself will continue to evolve as the therapeutic landscape shifts toward KRAS-targeted inhibitors and neoantigen vaccines, whose distinct mechanisms and timelines may produce patterns of regression, stability, and biomarker kinetics that current criteria were never designed to capture. Refining how treatment effect is measured—and revalidating those measures against each new class of therapy—will sharpen patient selection for surgery and improve prognostication in the modern neoadjuvant era for PDAC.
